# Revisiting consistency with random utility maximisation: theory and implications for practical work

**DOI:** 10.1007/s11238-017-9651-7

**Published:** 2018-01-02

**Authors:** Stephane Hess, Andrew Daly, Richard Batley

**Affiliations:** Institute for Transport Studies and Choice Modelling Centre, University of Leeds, Leeds, UK

**Keywords:** Random utility maximisation, RUM properties, Behavioural patterns, Discrete choice

## Abstract

While the paradigm of utility maximisation has formed the basis of the majority of applications in discrete choice modelling for over 40 years, its core assumptions have been questioned by work in both behavioural economics and mathematical psychology as well as more recently by developments in the RUM-oriented choice modelling community. This paper reviews the basic properties with a view to explaining the historical pre-eminence of utility maximisation and addresses the question of what departures from the paradigm may be necessary or wise in order to accommodate richer behavioural patterns. We find that many, though not all, of the behavioural traits discussed in the literature can be approximated sufficiently closely by a random utility framework, allowing analysts to retain the many advantages that such an approach possesses.

## Introduction

Discrete choice models have established themselves as an important tool for the analysis of individual decision making across numerous fields (see Anderson et al. 1992; Train 2009, for comprehensive overviews). The normative^1^ paradigm of utility maximisation has served as the basis for the vast majority of discrete choice models reported in the literature and, as we shall discuss, there are good reasons why this should be so.^2^ A historical perspective on this is given by McFadden (2000). Nevertheless, applications of positivist^3^ behavioural paradigms that depart from utility maximisation, or for which consistency with utility may be tenuous, have become more numerous and have been shown to represent aspects of behaviour that cannot be straightforwardly explained by utility (e.g. Chorus 2010; Leong and Hensher 2015; Guevara and Fukushi 2016). In this paper we, therefore, attempt to explore the basis on which utility maximisation is adopted, what behavioural phenomena have been detected that seem to be inconsistent with that paradigm and the issues that result from attempting to represent those phenomena in practical choice models.

The next section of the paper describes the random utility modelling (RUM) paradigm and how it has been used to model choice. This is followed by a discussion of behavioural ‘anomalies’ and an overview of efforts to accommodate these in choice models, looking both at extensions to RUM as well as the use of other model frameworks in this context. We highlight how some of these alternative structures actually remain close to RUM, while also questioning whether some of the extensions of RUM lead to violations of utility maximisation.

## The use of random utility to model choice

The concept of utility is fundamental to the standard microeconomic theory of consumer behaviour, where consumers are represented as choosing among bundles of continuously variable quantities of goods. Discrete choice might be seen as a variation on the standard theory, where consumers are represented as choosing the single alternative that, from a finite, exclusive and exhaustive set, maximises their utility, conditional on the constraints affecting the agent, in particular the budget.

In practical implementations of choice modelling, it is necessary to admit that the analyst does not know all, or indeed perhaps most, of the relevant facts about the agent to be modelled, such as the agent’s preferences, attitudes, income, exact location, etc. In particular, the process by which an agent makes choices is unknown to the analyst, and a paradigm such as utility maximisation is likely to be only an approximation to the real process used. Moreover, agents’ behaviour is likely to vary, systematically and/or idiosyncratically. For these reasons, practical models based on most paradigms contain a random element, such that choice is represented as a probabilistic phenomenon. This representation acknowledges the existence of uncertainties, but nevertheless allows the development of rigorous models. Issues of identification may, however, arise, so that it is often impossible to attribute randomness between the agent and the analyst: for example, perception error by the agent may be impossible to identify separately from measurement error by the analyst.

In this section, we first provide an overview of the history of random utility in choice modelling, before talking about the properties and benefits of the paradigm.

### The introduction of random utility to choice modelling

The idea of combining a random element with the concept of utility maximisation is intuitively appealing. Such models are generally referred to as RUM, a concept proposed by Marschak (1960) and Block and Marschak (1960), drawing from earlier work in the field of psychophysics (Fechner 1859). Formally, if we consider an agent making a choice from a finite set, random utility implies that there is a random vector $\left( {U_1,\ldots ,U_J } \right)$ unique up to an increasing monotone transformation such that the probability of choosing any alternative $i\in M$ (where *M* is the choice set) is given by:

$$\begin{aligned} P_i =\Pr \left( {U_i >U_j } \right) \quad \hbox { for all} \quad i\ne j\in M. \end{aligned}$$

As originally conceived, RUM was a ‘distribution free’ model (e.g. Regenwetter et al. 2010), in the sense that utility was conceptualised as a random ordinal variable, but without specific distributional properties. The development that completed RUM’s journey from theory to practice was to reconceptualise utility as a cardinal variable, comprising a deterministic element which is observable to the analyst, and a random element which is unobservable to the analyst and is typically referred to as the ‘error’ term. By assigning a distributional form to the latter, a ‘parametric’ version of RUM was developed that readily lends itself to implementation in practical policy and planning studies (McFadden 1968, but unpublished until 1975).

In some but not all paradigms, it is also admitted that agents may exhibit random behaviour. Block and Marschak saw RUM as a model of an individual agent engaged in a discrete choice task, whereby randomness arises from variations in choice behaviour across independent repetitions of that task. Prompted by the interest of economists in aggregate behaviour, McFadden re-interpreted RUM as representative of a population of decision-makers with explicitly varying tastes, each facing a single discrete choice task. McFadden (1981) argued that the two interpretations are formally equivalent, i.e. that the probability of a choice made by a specific individual in (conceptually) repeated experiments also applies to the probability of that choice made by a randomly chosen individual in an extensive population. Of course, we may in practice have individuals with both differing tastes and random behaviour.

The primary motivation of Marschak and his associates was to formulate models that could help in understanding choice processes. In particular, they sought to confirm through experimental investigation the propensity of decision-makers to adhere to RUM (e.g. Davidson and Marschak 1959), or at least to choice behaviour consistent with RUM. They did not seek to relate choice to characteristics of the alternatives and/or decision-maker, a development which followed some years hence.

Whereas the conventional microeconomic definition of ‘direct’ utility expresses utility as a function of the quantities of goods consumed, the developing discrete choice paradigm powerfully exploited Lancaster (1966) re-conceptualisation of goods in terms of their constituent attributes (potentially encompassing a range of ‘quality’ variables). In this way, modellers were able to link observations of choice to the quantities of various qualitative attributes characterising the available alternatives. Borrowing from the microeconomics of duality theory, choice modellers also drew upon the concept of ‘indirect’ utility, to introduce prices and incomes alongside quality variables, and this opened the door to two strands of model development and application. First, choice modelling became established as a key method for non-market valuation, since it could be used to examine marginal rates of substitution between quality variables and money, thereby eliciting marginal valuations of the quality variables with money as the numéraire. Second, through strengthening the theoretical grounding of choice models in terms of welfare economics, marginal valuations could be aggregated across both quality variables and individual decision-makers, to yield societal level valuations of policy or planning interventions.

In early practical applications of RUM for choice, marginal valuations of quality variables—specifically the willingness to pay for reductions in travel time—were elicited as a by-product of McFadden (1973) study, but as the principal product of Daly and Zachary (1975) study. Societal valuations were pioneered by McFadden (1978). RUM choice was also exploited in the forecasting of behaviour by Domencich and McFadden (1975), Williams (1977) and Daly and Zachary (1978). Some of these papers from the 1970s made significant theoretical contributions, which were subsequently consolidated within a more comprehensive welfare framework by McFadden (1981), Small and Rosen (1981) and Hanemann (1982). Further methodological developments (especially in the shape of more specific econometric specifications of RUM) and innovative applications then followed across numerous disciplines, especially in transport.

Thus, for more than 40 years RUM has been applied to serve the two main practical objectives of choice modelling: forecasting behaviour and extracting valuations, both as individual willingness to pay and as societal welfare calculations. RUM has also been used as a tool to investigate the fundamentals of behaviour, but it is in this latter role that it has been most strongly challenged by alternative paradigms or generalisations. Whilst acknowledging possible differences in interpretation—depending on the normative or positivist dichotomy—these objectives of RUM practice require that we must have a model that is ‘representative’ of agents’ behaviour. This assumption, albeit very strong, is essential to give credibility to model applications (Daly 1982). A key distinction arises here between the model offering an accurate representation of the choice process (which is not observed) and an accurate prediction of the choice outcome (which is observed). It is important to note that the latter may not necessarily require the former, but that the criticisms of the framework have focussed on the representation of process.

In the initial conceptualisations of RUM by Marschak and colleagues, and by McFadden (1973), the concept of RUM was introduced by construction. That is, the model was specified by defining a utility a priori. Randomness was then introduced by making the utility random or by making behaviour a random function of fixed utility (see, for example, Busemeyer and Rieskamp 2014). The ‘fixed utility’ approach can always be emulated by adding an appropriate random component to the utility, and for this reason models with an explicitly random utility have been much more prevalent. It is important to note that the inclusion of a random element is a generalisation rather than a restriction of the general behavioural framework. Trivially, if the variance of the random element becomes very small, the influence of random effects in the model becomes negligible. Moreover, the use of the RUM paradigm is an extremely general approach to modelling behaviour, providing that individual behaviour at each moment is consistent with utility maximisation, or that deviations are small enough to be allowed for by the random component of RUM. Again we note that RUM may be able to accurately or closely predict outcomes of behaviour that may not be completely consistent with utility theory.

### Characterisation of RUM

Given a specification of a model in terms of the distribution of the random utilities, it is theoretically straightforward to calculate choice probabilities by integration, and welfare by further integration. These provide key benefits of the paradigm, a point that we return to in the next section. Of course, the practical calculation of these measures can present moderate or severe difficulty in the more complicated cases, especially where income effects of price and/or income changes are non-linear. However, subsequent to the initial constructive RUM specifications, analysts began to ask whether models specified in terms of probability statements or welfare functions could be tested for consistency with RUM.

In this regard, it is useful to return to our earlier distinction between ordinal and cardinal (or parametric) RUM. With regard to ordinal RUM, it is well established that for binary choice probabilities involving up to five distinct alternatives, RUM holds if and only if the so-called ‘triangle inequalities’ hold (Cohen and Falmagne 1978; McFadden and Richter 1970a, b, 1991; Fishburn 1998; Cavagnaro and Davis-Stober 2014), i.e.

for any three distinct elements $x,y,z\in M$(where *M* is the choice set), the binary choice probabilities $P\left( \cdot \right)$ of preferring one alternative over another are given by:

1 $$\begin{aligned} 1\le P\left( {x,y} \right) +P\left( {y,z} \right) +P\left( {z,x} \right) \le 2 \end{aligned}$$

With regard to cardinal RUM, a fairly comprehensive test in terms of probability statements is given by Zachary’s theorem (Daly and Zachary 1978; proof given in Zachary 2012), whilst McFadden (1981) defined a class of models—termed Generalised Extreme Value (GEV) models—that could be shown to be RUM-consistent on the basis of their welfare functions. More recently, Fosgerau (2013, definition 1) have defined RUM by a cumulative distribution of utilities that is absolutely continuous, so that choice probabilities can be obtained. This recent work, which builds on the key earlier contributions mentioned above, may be taken as a definitive statement of cardinal RUM. However, it is notable that Definition 1 in Fosgerau et al. (2013) specifically excludes issues of economic rationality. These issues are introduced later in the paper where, among other requirements it is specified that utility “may depend on other variables [...] that include attributes of the discrete alternative and factors that influence tastes, such as age and family size”. A requirement that the utility of one alternative should not depend on the attributes of another alternative is implicit but not stressed. Another key contribution was made by McFadden and Train (2000), who showed that a fully specified Mixed Logit model is the most general structure and can approximate any RUM arbitrarily closely. A seemingly ignored fact is that the same point was made earlier by Dalal and Klein (1988).

From these contributions, a series of tests can be derived that allow practical models to be assessed in terms of RUM compatibility and other related properties. Central among these tests is regularity (Marschak 1960, p. 192), whereby the probability of choosing any given alternative from an offered set should not increase if the offered set is expanded to include additional alternatives. Regularity follows from the assumption that choice is based on utility maximisation, i.e. it is a necessary (though not sufficient) condition.

Another test which has attracted considerable interest is transitivity, whereby if alternative A is preferred to alternative B, and B to C, then alternative A should be preferred to alternative C. Recognising that transitivity is ostensibly a deterministic property, the discrete choice literature has developed various stochastic interpretations of transitivity (referred to as ‘weak’, ‘moderate’ and ‘strong’), which relate to the probability that alternative A is preferred to alternative B, and so on. Whilst none of these interpretations are necessary for RUM, there is a close relationship between stochastic transitivity and the triangle inequalities.

Suppose that we can view an individual’s discrete choice as resulting from a complete and transitive preference structure that gives an ordering of preference of the alternatives. At any moment this ordering is clearly defined but could be the result of a random process, e.g. it may vary over successive choices by that individual. Then this preference structure is consistent with utility maximisation (Block and Marschak 1960) and hence preferences are regular, in the sense that the ordering among the initial set of alternatives would not be disrupted by the addition of further alternatives to the set. In other words, for a given individual at a given moment, utility maximisation implies regularity. Integrating over the preference structures, we obtain the result that irregularity by an individual implies a failure of utility maximisation by that individual at some moments and that irregularity in a population implies a failure of utility maximisation by some individuals at some moments.

Since regularity is necessary, RUM implies that the utility difference of any pair of alternatives may not depend on the attributes of another alternative. Dependence of the utility difference on the attributes of another alternative can easily bring about a failure of regularity,^4^ as a change in the attributes of alternative C may affect the instantaneous preference (or the aggregate choice probability) between alternatives A and B. The key feature of utility maximisation in this context is that by choosing an alternative, the agent enjoys the attributes of that alternative, without reference to the attributes of any other alternative.

Several of the most interesting behavioural paradigms introduced in recent years relate the utility of an alternative to its relationship to other alternatives, as discussed in the following section, but these paradigms are then in some cases clearly inconsistent with utility maximisation, with all the consequences that follow from that inconsistency. Consistency with utility maximisation is not achieved by most implementations of the Mother Logit model (McFadden 1975), nor by popular recent developments such as Random Regret Minimisation (RRM; see, e.g. Chorus 2010). Furthermore, for consistency with utility maximisation the sign of the utility difference between any two alternatives should not be affected by the addition/subtraction of alternatives to/from the choice set. This is subtly (but importantly) different from the ‘invariance’ property (e.g. Batley and Hess 2016), where the choice probability between two alternatives is invariant to addition/subtraction of alternatives; this is a property of Multinomial Logit (MNL), but not RUM in general.

The situation concerning reference dependence, where choice is affected by features established prior to the choice to be modelled, is different. Here, the reference situation is part of the agents’ preference structure, and the dependence of the utility of alternatives on features of the reference situation does not affect the consistency of the model with RUM. Note that this applies even where reference is defined with respect to a *status quo* alternative and the choice set includes the *status quo* alternative, as the function of that alternative in defining the agents’ preference structure is distinct from the possibility that the status quo alternative may continue to be chosen.

The most interesting RUMs are of course those in which utility can be represented as a function of the attributes of the alternatives and conditioned by the characteristics of the agent. As noted above, by focusing on indirect utility, these may include the price of each alternative and the income of the agent. However, in an analogous manner to the more conventional economic context of continuous consumption, the implementation of indirect utility within discrete choice contexts encounters the classic Marshallian problem of heterogeneity in the marginal utility of income and the associated issue of path dependence (e.g. Batley and Ibanez 2013). Maintaining the analogy to continuous consumption, recognition of this problem has prompted some RUM researchers to adopt the standard Hicksian solution to path dependence (e.g. Hau 1985; Karlström and Morey January 2001; Dagsvik and Karlström 2005), which is essentially to convert the numéraire from utility to money. Unfortunately, this literature has been slow to develop, and contemporary random *utility* modelling would seem committed to a Marshallian framework.

Within this Marshallian framework, the dependence of utility on attributes naturally leads to the specification of a cardinal utility measure, on which conditions arising from economic theory may be imposed. For example, it cannot be the case that the price of an alternative has a positive influence on its utility (unless price is operating largely as a proxy for quality variables). In addition to these economic tests, conditions may be applied that arise from behavioural considerations. For example, sign conditions or relative value conditions may be applied to the values of estimated coefficients. Testing models in this way can make a valuable contribution to obtaining models that give good results for whatever objectives the modelling may have.

In summary, by RUM we mean a model in which each alternative has a cardinal or ordinal utility, expressed in a common numéraire; utility is not known in full by the analyst but is capable of approximation with error. The model must comply with the triangle inequalities, must not exhibit preference reversal and, therefore, the utility differences of any pair of alternatives must not depend on the characteristics or existence of another alternative.

### Benefits of RUM

The use of the RUM paradigm brings substantial benefits, so that constraining choice models to that paradigm, where possible, is often found to be beneficial, even though there may be a loss in explanatory power or in the clarity of theoretical exposition of the model. In the following section we discuss how some behaviour that is at first sight ‘non-RUM’ may be approximated by RUM, thereby retaining the benefits of the RUM approach.

It is important to note that consistency with RUM does not necessarily imply that behaviour arises from individuals assessing the attributes of the alternatives they face, deriving utilities and choosing the best-performing alternative. Instead, it is postulated that behaviour that follows the rules outlined above may be characterised ‘as if’ behaviour followed RUM and the benefits of the RUM approach then follow. According to this interpretation, RUM could encompass a whole range of behavioural processes employed in practice, provided these can be reconciled in some shape or form with utility maximisation subject to constraints. Such behaviour is often described as ‘rational’,^5^ but it is debatable whether this characterisation is helpful to the discussion.

The key benefit of the RUM approach to the study of choice is the link it gives to microeconomics. Here there is a large body of theory and empirical evidence offering methodology and tests of behaviour. Setting the modelling within such a widely accepted behavioural framework helps in gaining acceptance for the approach, by providing a well-developed discussion of its strengths and weaknesses. For example, while microeconomics provides a sound basis for welfare analysis at the level of the individual, it also draws attention to the difficulty of integrating the measures over a population and presents plausible ways in which this might be done. It also provides a theoretically acceptable basis for the estimation of willingness-to-pay measures as marginal rates of substitution between the price and other attributes of alternatives. Similarly, for forecasting, RUM offers a credible basis that justifies expectations that individuals may continue to behave in ways that have been observed to date.

However, there are numerous examples in the literature of criticisms of the RUM approach, and interest in behavioural features that are not compatible with generally understood interpretations of RUM. In the following section we discuss a number of these apparent departures in detail, addressing the key issue of whether extensions or adaptations to RUM could accommodate or approximate these features, some more subtle than others. Clearly, our preference is to accommodate more behavioural realism while remaining, if possible, within the RUM framework with all its advantages.

Two general extensions may be mentioned here. First, it is reasonable to use models that may be consistent with utility maximisation only within a defined region; provided that no investigations need to be made outside this region, such models can claim the advantages of RUM. This would apply, for example, to models in which the impact of variables such as price changed sign outside the defined region (as would happen with specific non-linear treatments), or where the structure of the model implied inappropriate elasticities outside the region (Börsch-Supan 1990; Herriges and Kling 1996; Batley and Hess 2016). Second, it is possible to incorporate within the modelling different behavioural paradigms (Hess et al. 2012) that allow for the possibility that an agent’s behavioural process is unknown, or is itself random. However, this second extension does not extend the scope of RUM because, in order for the overall model to be RUM-consistent, it is necessary for each of the components to be RUM-consistent.

## Behavioural ‘anomalies’ and their treatment in choice models

From a positivist standpoint, a key criticism of utility-based models in the behavioural economics and mathematical psychology literature (e.g. Kahneman 2003) has been its inherent assumptions of so-called ‘rational’ behaviour which seems to contradict many findings from real world observations. However, as already mentioned in Sect. 2, consistency with RUM does not require agents to behave in a RUM-style process, only in one that yields choices consistent with it. Similarly, this literature seems to equate the term ‘rational’ with behaviour consistent with utility maximisation, which is not necessarily helpful.

There is ample evidence showing that in many contexts, agents’ judgements, preferences and behaviour are at face value systematically irrational (e.g. Kahneman and Tversky 1979; Tversky and Kahneman 1974). An important point to make at the outset is that a decision-maker’s behaviour might be considered rational from the perspective of that person; what irrational behaviour refers to is a lack of consistency with behavioural paradigms and rules as set out by the observer or analyst. It is here that the strong assumptions underlying some of the modelling frameworks can lead to problems.

Key findings include the following: individuals’ preferences and judgements are unstable and context dependent (Tversky and Simonson 1993; Ariely et al. 2003; Huber et al. 1982), individuals are cognitively constrained (Jamasb and Pollitt 2005) and individuals tend to use different cognitive procedures and rules to deal with complex decision problems (Tversky and Kahneman 1974; Manzini and Mariotti 2007). It should be acknowledged that these ‘findings’ are themselves often based on specific experimental settings which are developed in such a way as to tease out these anomalies (e.g. thinking of the zero cost example we discuss in Sect. 3.2.1) and these settings may in fact overstate the extent of such behaviour, particularly in real-world contexts.

It should also be noted that the criticisms raised in these literatures seem to refer to the utility maximisation paradigm without recognising that the move to *random* utility maximisation is in large part motivated by a desire to capture the types of inconsistencies and idiosyncrasies in behaviour discussed above. Indeed, the field has proactively sought to address some of these concerns, through refinements of and extensions to the set of RUMs. This goes back for example to discussions in Ben-Akiva et al. (1999), a paper which seeks to “discuss the consequences of various ‘anomalies’ of preference elicitation”. This key paper later led to the growing use of hybrid choice structures (see the extensive overview in Abou-Zeid and Ben-Akiva 2014), an approach that has, however, been exploited primarily for accommodating attitudes and perceptions in decision making, rather than some of the behavioural traits we discuss below. Crucially, Ben-Akiva et al. (1999) does not seem to have stemmed the interest in departures from RUM to accommodate such anomalies.

A key question we shall return to later in this section is, on the one hand, to what extent these ‘improvements’ may actually lead to violations of key RUM assumptions, and on the other hand, how different from RUM structures the new models actually are. Before turning to the individual phenomena, we focus on the general notion of context dependence, which is of key interest in behavioural economics and mathematical psychology and encompasses many of the issues covered in Sect. 3.2.

### Context dependence and RUM

We start our discussion by defining the utility that agent *n* obtains from choosing alternative *j* in choice situation *t* as $U_\mathrm{jnt}$, where, in a random utility framework, this is made up of a ‘deterministic’ component $V_\mathrm{jnt}$ and a random component $\varepsilon _\mathrm{jnt}$. This deterministic component is defined as $V_\mathrm{jnt} =g\left( {\beta ,x_\mathrm{jnt} ,z_\mathrm{n} } \right)$, where $\beta$ is a vector of estimated parameters, $x_\mathrm{jnt}$ are attributes describing alternative *j* as faced by agent *n* in choice scenario *t*, and $z_\mathrm{n}$ are characteristics of agent *n*. Returning to our earlier discussion concerning sources of randomness in Sect. 2.1, note that this framework admits both multiple agents and multiple repetitions of a given choice task.

Notwithstanding recent developments in multiplicative error structures (Harris and Tanner 1974; Fosgerau and Bierlaire 2009), the typical assumption of an additive error structure means that the probability of agent *n* choosing alternative *i*(out of $j=1,\ldots ,J)$ in choice task *t* is then given by:

2 $$\begin{aligned} P_\mathrm{int} =P\left( V_\mathrm{int} +\varepsilon _\mathrm{int} >V_\mathrm{jnt} +\varepsilon _\mathrm{jnt}, \quad \forall j\ne i\right) . \end{aligned}$$

As is well known, with an assumption that the error terms $\varepsilon _\mathrm{jnt}$ are independently and identically distributed (*iid*) according to a type I extreme value (EV1) distribution, this leads to a Multinomial Logit (MNL) model, with:

3 $$\begin{aligned} P_\mathrm{int} =\frac{e^{V_\mathrm{int} }}{\mathop \sum \nolimits _{j=1}^J e^{V_\mathrm{jnt} }}. \end{aligned}$$

With more flexible specifications of the error terms, we can move to other members of the family of Generalised Extreme Value (GEV) models (if there is correlation across some alternatives in $\varepsilon _\mathrm{jnt} )$, Mixed Logit models (in the presence of additional random error on top of the *iid *EV1 errors) or Probit models (if $\varepsilon _\mathrm{nt}$ follows a multivariate Normal distribution).

These departures from the most basic assumption about the error structure can lead to important gains in model performance and may in fact allow the model to accommodate some of the behavioural phenomena that are central to the discussions in behavioural economics and mathematical psychology without explicitly describing them. This is in line with the theoretical discussions in McFadden and Train (2000) and the empirical results in Hess et al. (2017). While this may not satisfy the desire for behavioural realism, it allows the model to represent the behaviour closely enough to produce good predictions while retaining other benefits inherent to RUM structures.

A key question that an analyst needs to consider in this context is which is most important; the explicit modelling of the behavioural processes or the retention of the microeconomic framework underlying RUM? This is strongly related to the application of the model for valuation and forecasting. If analysts wish to retain the framework of (2) but explicitly model specific behavioural phenomena that cannot be accommodated in the error structure, then attention inevitably turns to the definition of $V_\mathrm{jnt}$. An important component in this is the impact that the context in which a choice is made has on outcome of the choice. As we will see in the later discussion, if the source of this impact is exogenous to the comparison between the alternatives, then consistency with RUM can generally be maintained. This is no longer generally the case when the context effects are driven by the choice set itself.

While interest in behavioural flexibility and realism has grown in recent years, it is important to remember the early efforts of McFadden (1975) in this context, specifically with regard to the development of the Mother Logit (or Universal Logit) model, which will serve as a useful ‘straw man’ against which subsequent developments in model form regarding context dependence can be assessed. McFadden (1975) introduced this model as:

4 $$\begin{aligned} P_\mathrm{int} =\frac{e^{g_\mathrm{int} }}{\mathop \sum \nolimits _{j=1}^J e^{g_\mathrm{jnt} }}, \end{aligned}$$

where $g_\mathrm{int} =f_i \left( {V_\mathrm{jnt} ,\forall j} \right)$, where no constraints are imposed on this function.

Note that the same model can be achieved by replacing $V_\mathrm{jnt} =g\left( {\beta ,x_\mathrm{jnt} ,z_\mathrm{n} } \right)$ in (2) with $V_\mathrm{jnt} =g\left( {\beta ,x_\mathrm{nt} ,z_\mathrm{n} } \right)$, where $x_\mathrm{nt}$ now contains the attributes of all alternatives in the choice set, allowing for rich patterns of context dependence to be incorporated. While McFadden (1975) initially highlighted the potential flexibility of the Mother Logit framework, McFadden et al. (1977) subsequently noted its general lack of consistency with utility maximisation, given the potential for failures of regularity. McFadden (2000) wrote: “ I called this the mother logit approximation, and suggested that it could be used as an alternative against which to test IIA. Because there was no easy way to tell whether a mother logit model was consistent with RUM, it did not provide a useful setup for estimating general RUM-consistent models or testing for RUM-consistency.” As we will see in what follows, those behavioural phenomena that need to be accommodated in a Mother Logit style functional form (albeit that this link is often ignored by authors) will lead to violations of RUM.

### Behavioural phenomena and their representation in models of choice

We will now provide a brief review of a number of key behavioural phenomena. This list is not meant to be complete and the inclusion of topics is unavoidably selective. Each time, we seek to discuss the behavioural relevance of the topic, the likely impact of not accommodating the phenomenon in our models, and an overview of attempts (if any) to represent the effect in the choice modelling literature. With regard to the last point, we specifically look at the implications of such efforts on maintaining consistency with utility maximisation. We group the phenomena together according to whether or not they are theoretically consistent with RUM.

#### Generally fully consistent

*Anchoring effects*

Anchoring effects refer to the phenomenon that individuals’ decisions could be affected by external cues. A crucial initial investigation came in the work of Tversky and Kahneman (1974), who demonstrated that students’ judgements of the percentage of African countries in the United Nations were biased towards a random number generated by a ‘wheel of fortune’. Since then, behavioural economists and psychologists have found salient and robust anchoring effects in both experiments and real world choices.

In the context of the choice modelling literature, the main focus on anchoring effects has been how a previous choice setting can influence preferences in a subsequent choice setting. A key example comes in value of time work, especially where based on stated choice data. If a respondent is faced with a choice in task 1 where he/she can *purchase* a reduction in travel time at a cost of £x/h, then this may influence his/her willingness to *purchase *a reduction at a cost of £year/h (where £y may be smaller or larger than £x) in subsequent tasks. Anchors may form specifically the first time a respondent faces a given type of choice, but subsequent choices may refine the anchor. The influence of anchoring on the value of time has been considered in some depth by VandeKaa (2005).

The specification of anchors may vary, and an anchor could be formed either by what a decision-maker ‘sees’ in a given choice task or by what he/she chooses. An anchor may also be constant (formed the first time a respondent faces a particular choice) or evolve over time (e.g. changing with each choice situation). If, in each choice situation, the choice is modelled with a RUM structure, then the actual choice is consistent with RUM, but the sequence is not consistent with a single definition of utility, as utility gets redefined over time, either just once for all choices following the initial choice, or after each choice. This is consistent with the original Block and Marschak (1960) interpretation of RUM. Either way, such heterogeneity in valuations over time is not in principle inconsistent with RUM.

*Zero cost/price effects*

In an example made famous by Dan Ariely’s book *‘Predictably Irrational’* (Ariely 2008), and based on Shampanier et al. (2007), individuals’ choices between two chocolate products changed substantially when an equal reduction in the cost (i.e. price) of the two products led to a zero cost for one of the two. Such effects are also visible in many stated choice surveys where one or more of the alternatives in a choice task have a zero cost to the respondent, be it in the case of toll road studies (e.g. Hess et al. 2008) or the numerous environmental economics datasets including a zero cost status quo alternative (see the discussion on confounding in Hess and Beharry-Borg 2012). The behaviour exhibited by this effect is not consistent with a linear cost sensitivity, which is a core assumption in many applications of choice models. However, it can easily be accommodated through a non-linear specification and does not lead to violations of utility maximisation.

*Status quo bias*

Status quo bias refers to the phenomenon that individuals have strong propensity to choose the alternative that describes their current situation. It was first demonstrated by Samuelson and Zeckhauser (1988), but is commonly observed in many stated choice surveys, especially when the status quo alternative is explicitly labelled as such. The fact that individuals attach undue weight to their current situation does not lead to any issues from a utility maximisation perspective, and is routinely accommodated in models. A different issue of course applies if these models are used in forecasting, where the status quo is unknown. Applications looking at this issue are common in environmental economics, see for example Meyerhoff and Liebe (2009).

*Mental accounting*

Mental accounting refers to the cognitive process by which individuals allocate their overall money budget into different mental accounts. It is a common empirical finding that money in one mental account is not a perfect substitute for money in another account, thus violating the principle of fungibility (Thaler 1985). This effect is commonly observed in transport choice models with multiple cost components (e.g. different responses to fuel costs and toll costs) and has for example been studied in a stated choice context by Hess et al. (2012). While this behavioural effect poses issues from an economic theory perspective, it does not pose any particular issues for a theoretical RUM-consistent model of choice behaviour.

*Elimination by aspects*

Elimination by aspects (EBA), which was proposed by Tversky (1972a, b), posits that an agent successively eliminates alternatives that fail to possess aspects that the agent finds necessary or important. Noting that the elimination process establishes a branching choice structure, several authors have suggested similarity with the nesting structures of McFadden (1978) RUM-consistent GEV model. This suggestion was investigated in detail by Batley and Daly (2006), who found that there was equivalence between ‘hierarchical’ EBA (where there is a unique sequence of eliminations to reach each alternative) and ‘tree’ Nested Logit models (where again there is a unique choice sequence). Although more general EBA and Cross-Nested Logit models are not necessarily equivalent, despite the apparent similarity of structure, Tversky (1972a) presented a much-neglected proof that, by re-interpreting EBA as a ranking model, general consistency between EBA and RUM can be established.

#### Not consistent in general or in some cases

*Lexicography and extreme sensitivities*

Lexicography refers to the case where, typically in an experimental setting, a decision-maker evaluates the alternatives on the basis of a subset of attributes (e.g. Sælensminde 2006). Common examples include respondents who always choose the cheapest alternative irrespective of the other attributes shown, or travellers who always choose the fastest alternative. Lexicography may also exhibit itself as non-trading if, for example, respondents always choose the same mode in a transport setting. This type of behaviour may be consistent with utility maximisation if it reflects true preferences, i.e. extremely high sensitivities to given attributes, such that a change in behaviour would arise only with a sufficiently large incentive. If, however, it is caused by strategic behaviour in a survey context, violations of RUM may arise. Lexicographic behaviour may also be the result of choice set complexity, leading to decision makers adopting processing heuristics, an issue we return to below.

*Reference-dependent preferences and loss aversion*

The topics of reference dependence and loss aversion are generally attributed to Tversky and Kahneman (1991) and have become a widely studied topic in choice modelling in recent years. The central argument is that when individuals evaluate their response to a given stimulus, i.e. the value of an attribute $x_\mathrm{jntk}$ (the *k*th component of $x_\mathrm{jnt} )$, this valuation depends not just on the absolute value of this attribute, but also on its value relative to a reference point, say $r_\mathrm{nk}$. For an undesirable attribute, respondents are expected to react negatively to increases in $x_\mathrm{jntk}$ and positively to decreases. When these reactions are symmetrical, we return to the standard specification, where the contribution to the utility of alternative *j* is given by $\beta _k x_\mathrm{jntk}$ (under the assumption of a linear specification). Loss aversion postulates that losses are more painful than gains are pleasurable, and we then instead have that the contribution is driven by separate loss ($\beta _\mathrm{k,loss} )$ and gain ($\beta _\mathrm{k,gain} )$ parameters $\beta _\mathrm{k,loss} \left( {x_\mathrm{jntk} -r_\mathrm{nk} } \right)$ if $x_\mathrm{jntk} >r_\mathrm{nk}$, and $\beta _\mathrm{k,gain} \left( {r_\mathrm{nk} -x_\mathrm{jntk} } \right)$ if $x_\mathrm{jntk} <r_\mathrm{nk}$, where we would expect that $\beta _\mathrm{k,loss} \le 0\le \beta _\mathrm{k,gain}$ and $\mid \beta _\mathrm{k,loss} \mid \ge \mid \beta _\mathrm{k,gain} \mid$.

Empirical support for reference dependence and loss aversion is widespread in the choice modelling literature (e.g. Hess et al. 2008) and has also led to the development of bespoke modelling approaches (cf. de Borger and Fosgerau 2008). What has received little or no attention is the impact on consistency with utility maximisation. With reference dependence, the utility of an alternative depends on the characteristics of the alternative and the reference point. It should be clear that if the reference point is independent of the composition of the choice task, then the inclusion of reference dependence in a model will not lead to a violation of utility maximisation. Indeed, the addition of an alternative into the choice set will not change the utilities of other alternatives, and the probabilities of all existing alternatives (prior to the new one being added) will not increase—thereby complying with regularity. This applies whether or not the reference alternative itself is included in the choice task, or indeed if the reference alternative is the alternative that is being added. If the reference point changes over time, then preferences will of course change too, but this is not a problem for utility maximisation. As a final point, if the reference alternative is included in the choice task, say as alternative 1, then a standard implementation of a model for such data (as in Hess et al. 2008) is in effect a Mother Logit structure, where, e.g. $g_\mathrm{int} =\mathop \sum \nolimits _k \left[ {\beta _\mathrm{k,inc} \cdot \max \left( {x_\mathrm{intk} -x_\mathrm{1ntk} ,0} \right) +\beta _\mathrm{k,dec} \cdot \max \left( {x_\mathrm{1ntk} -x_\mathrm{intk} ,0} \right) } \right]$, where *k* is an index over attributes. This is thus an example where a Mother Logit structure is consistent with utility maximisation, as the utility for alternative *i*is only a function of its own attributes and the fixed attributes of the reference alternative. Effectively, the reference alternative becomes part of the preference structure at the moment of choice, and the alternatives are evaluated in that preference structure using only their own attributes.

*Decoy, context and framing effects*

The term ‘decoy effects’ has been used to describe a set of slightly different effects, including asymmetric dominance effects, attraction effects, compromise effects and phantom decoy effects. Asymmetric dominance effects were first described by Huber et al. (1982), who found that in a binary choice task, adding a third alternative (i.e. decoy) that is dominated by one alternative but not the other can shift individuals’ preferences towards the alternative that dominates the decoy. An attraction effect (Huber and Puto 1983) arises when the decoy is ‘nearly dominated’ rather than ‘fully dominated’ by one alternative in the choice set but not the other, i.e. if it is outperformed by one alternative on all its characteristics except one, where it only has a small advantage for the latter. A further possibility is that of a ‘phantom decoy’ effect (Pratkanis and Farquhar 1992), where the decoy can be ‘seen’ but is unavailable for choice. Finally, in a compromise setting, the decoy is not dominating or dominated by any alternative, but has a combination of small advantages and disadvantages in relation to the other alternatives. Such compromise alternatives can have increased probability of being chosen when individuals are averse to extreme outcomes.

Decoy effects in discrete choice modelling have been studied by Guevara and Fukushi (2016) and Rooderkerk et al. (2011), as well as by Chorus and Bierlaire (2013) in the context of compromise effects. The presence of decoy alternatives will lead to changes in the relative probabilities of other alternatives and, with the exception of the phantom decoy which cannot be chosen, their inclusion in the choice set has the potential to lead to an increase in the probability of one or more alternatives; this breaches regularity and makes such effects inconsistent with RUM.

Context effects cover a broader range of issues that relate to the fact that the relative choice probabilities across alternatives may differ depending on the presence or absence in the choice set of other alternatives. They cover attraction, compromise and similarity effects, some of which can also be classified under the decoy points above. Similarity effects are at the heart of the development of nested logit structures in choice modelling. If the effect is captured purely through the error structure of the model, and if specific conditions on the nesting structure are satisfied (Batley and Hess 2016), then the model remains consistent with utility maximisation.

Problems arise when the cross-substitution effects are captured through the observed component of utility, since the size and sign of associated coefficients can lead to preference reversals. Examples in the mainstream choice modelling literature include models used for route choice behaviour, where the impact of the overlap of different routes is captured in the observed utility component. Two popular examples are the C-Logit model developed by Cascetta et al. (1996) and the path-size approach of Ben-Akiva and Bierlaire (1999a). Both approaches include in the utility function of alternative *i* a measure of the similarity/overlap with other alternatives ($j\ne i)$ and thus open up the possibility of preference reversals as this component depends on the attributes of other alternatives in the choice set (again in the manner of Mother Logit) and changes in the composition of the choice set.

Framing effects refer to the phenomenon that individuals’ judgements and decisions could be affected by changes to the descriptions of the same piece of information. Framing effects violate the normative principle of description invariance (Tversky and Kahneman 1981), but do not affect consistency with utility maximisation.

*Regret*

Loomes and Sugden (1982) put forward the notion that an individual’s utility is not only derived from the chosen alternative but also from the regret or the ‘rejoicing’ generated from the differences between the chosen alternative and the alternative he/she forgoes.

Regret has received widespread attention in choice modelling in recent years, with the development of successive versions of a Random Regret Minimisation (RRM) framework (cf. Chorus 2010).

In the most widely used implementation (Chorus 2010), the regret associated with alternative *i* in choice task *t* for agent *n* is instead obtained as:

5 $$\begin{aligned} R_\mathrm{int} =\sum \nolimits _{j\ne i} \sum \nolimits _k \ln \left( {1+\hbox {exp}\left[ {\beta _\mathrm{k} \cdot \left( {x_\mathrm{jntk} -x_\mathrm{intk} } \right) } \right] } \right) \end{aligned}$$

where *k* is an index of attributes. With the assumption of a type I extreme value error and the notion of regret minimisation rather than maximisation, we then have (with either (2) or (3)) that:

6 $$\begin{aligned} P_\mathrm{int} =\frac{\hbox {exp}\left( {-R_\mathrm{int} } \right) }{\mathop \sum \nolimits _{j=1..J} \hbox {exp}\left( {-R_\mathrm{jnt} } \right) }, \end{aligned}$$

It can clearly be seen from (5) that the RRM model is in fact a specific version of a Mother Logit model, with the utility of an alternative depending on the attributes of other alternatives, where $g_\mathrm{int} =-\mathop \sum \nolimits _{j\ne i} \mathop \sum \nolimits _k \ln \left( {1+\hbox {exp}\left[ {\beta _k \cdot \left( {x_\mathrm{jntk} -x_\mathrm{intk} } \right) } \right] } \right)$. RRM is thus not a *novel* type of model but remains a Logit model, albeit one that, like most Mother Logit specifications, is not consistent with utility maximisation. While this lack of consistency has been acknowledged by authors using RRM, and indeed seen as an advantage, this link with Mother Logit has not previously been made to the best of our knowledge. A special case arises when $J=2$, where RRM is formally equivalent to a RUM-consistent Logit model with (5) (cf. Chorus 2010). With RRM, it is easy to see how the inclusion of an additional alternative can increase the choice probability of one or more of the alternatives already in the choice set, i.e. the model does not exhibit regularity, given that the regret needs to be recalculated for all alternatives in the choice set.

*Complexity, simplification of choice tasks and heuristics*

A number of authors have addressed the issue of choice complexity, especially in the context of stated choice surveys (e.g. Rose et al. 2008). These papers have looked at the impact that the composition of the choice environment, in terms of number of alternatives, attributes and attribute levels, has on the level of noise in the data (i.e. model scale) as well as substantive outputs (e.g. willingness-to-pay measures). At the same time, there is a growing literature in choice modelling looking at how individual decision-makers process the information describing the choices they face and what heuristics they may use (e.g. potential attribute ‘non-attendance’). Other work has looked at the role of choice set generation, where individuals may look at only a subset of the available alternatives (e.g. Manzini and Mariotti 2014).

The majority of the above work has been conducted with the use of random utility models. The focus has generally been on behaviour within a given context and by a given person, e.g. making the heuristic specific to a given individual. However, if one makes the link between the literature on choice task complexity and the literature on choice process, then it is clear that the presence of such effects may in fact lead to violations of key principles of utility maximisation. As an example, if the inclusion of additional alternatives into a choice set changes the way in which respondents make their choice, i.e. leading to the application of a *different* RUM, and if this effect differs across alternatives (due to differing attribute values), then the potential for preference reversals clearly exists, as the utility functions become dependent on attributes of other alternatives. On the other hand, it is also worth noting the existence of work looking at the role of inattention (which can link to complexity) and incorporating this in an Additive Random Utility Model (ARUM) context (cf. Matejka and McKay 2014).

### Overview and potential for forecasting and welfare analysis

Table 1 summarises the RUM-consistency of the specific behavioural phenomena discussed in Sect. 3.2 above, and each time gives a key recent reference in choice modelling. The table also indicates whether these phenomena can be incorporated into forecasting, valuation and welfare analysis. In forecasting, there is inevitably consideration of a changed situation, which may be described as ‘do-something’ (i.e. a price or quality change), and this may or may not involve a significant time difference. While the computation of marginal willingness-to-pay estimates may be possible, welfare analysis always involves a price or quality change, giving rise to a comparison of do-something against do-nothing.

We see that, in most cases, the phenomena can be represented within a RUM-consistent framework estimated for the do-nothing situation (the status quo scenario), and this makes basic valuation calculations possible in the situation where no major changes to the choice scenario arise. However, in many cases, it is difficult or impossible to include them in forecasting, because it is far from obvious how the phenomena would translate to the do-something situation (i.e. where a significant change is made to one or more of the alternatives/attributes). This is of course more particularly likely to happen when there is a significant time difference between do-nothing and do-something situations. In even more cases, welfare analysis is difficult or impossible, because of the additional requirement to aggregate measures of welfare change under the do-something scenario across alternatives and individuals, leading to further non-linearities.

**Table 1:** Summary of key behavioural phenomena, their consistency with RUM, and their practicability for forecasting and welfare analysis

	Theoretical RUM-consistency	Forecasting	Willingness to pay calculations	Welfare analysis	Example choices modelling references
Anchoring	Yes	May be impossible, because anchors cannot be forecast	Possible, but asymmetry and potential non-linearity cause further complications	May be impossible because of inability to forecast	VandeKaa (2005)
Zero cost bias	Yes	Yes	Possible in principle, but potential for extreme measures of welfare in practice		No applications so far
Status quo bias	Yes	No	Possible	Impossible because of inability to forecast	Meyerhoff and Liebe (2009)
Mental accounting	Yes	Perhaps	Possible	Difficult or impossible because of inconsistent numéraire	Hess et al. (2012)
Elimination by aspects	Yes	Yes	Yes for nested case, otherwise not; also a risk of extreme welfare effects		Batley and Daly (2006)
Lexicography	Not if caused by strategic behaviour	May be impossible if lexicography is a survey artefact	Possible but not if denominator is affected	Difficult or impossible, because of potential for different lexicographic effects across alternatives and individuals and extreme welfare measures	Sælensminde (2006)
Reference dependence	Some forms of reference dependence imply loss of consistency	Only in neighbourhood of the do-nothing, as reference point would otherwise be likely to change	Possible, but asymmetry and potential non-linearity cause further complications	Difficult or impossible, because of potential for different references across alternatives and individuals	Hess et al. (2008)
Decoy, context, framing	Only in some cases	No, because context cannot be forecast	Impossible in general as becomes choice set dependent	Impossible in general, except in binary case	Guevara and Fukushi (2016)
Regret	Not if more than two alternatives	Only in neighbourhood of the do-nothing	Impossible in general as becomes choice set dependent, except in binary case	Impossible in general, except in binary case	Chorus (2010)
Simplification	May not be	May be impossible if simplifications are not constant	Possible	May be impossible if simplifications are not constant	Rose et al. (2008)

However, in some cases, e.g. models using the de Borger and Fosgerau (2008) formulation, some of the reference-dependent effects can be eliminated for willingness-to-pay calculations

## Conclusions

The paradigm of utility maximisation has underpinned the vast majority of discrete choice models reported in the literature, and there are good reasons why this is so, since consistency with RUM greatly improves the applicability of models for forecasting and economic valuation at both individual and societal levels. However, there has also accumulated a comprehensive literature documenting real-world and/or experimental choice contexts that validate alternative behavioural paradigms, and where RUM seemingly offers a poor description of actual behaviour. Against this background, the present paper explored the basis on which RUM is adopted, what the alternative approaches might be and the relative advantages of the various approaches.

Our main conclusion is that the RUM paradigm has served choice modelling well. If we adopt a normative perspective then RUM can in principle admit a whole range of behavioural processes employed in practice, provided these are reconcilable in some shape or form with utility maximisation subject to constraints. On this basis, RUM is rather more flexible and agile than might appear from a positivist perspective, since many of the behaviours which have been promoted as ‘non-RUM’ in the literature can be recast as RUM-consistent or can be approximated by RUM-consistent models. That said, there remain some behaviours which are intrinsically non-RUM: these imply behavioural features such as irregularity, non-transitivity or preference reversal.

We illustrated the above conclusion in relation to several behavioural phenomena which have attracted particular interest in real world and experimental studies (summarised in Table 1). Arising from this illustration, three considerations are perhaps pertinent:

- First and foremost, whether or not a given phenomenon is RUM-consistent—the key violations here are certain forms of reference dependence and simplification heuristics.
- Second, the practicability of forecasting the phenomenon under the do-something—this is challenging for many of the phenomena because of their context specificity to the do-nothing, and the difficulty of transferring the phenomena to the do-something.
- Third, the challenges of forecasting follow through into welfare analysis—here they are compounded by additional difficulties associated with variations in the behavioural phenomena across alternatives and individuals.

Our concluding message is that the developments in both the scientific and non-specialist literature on behavioural economics have certainly revitalised the field of choice modelling, raised a number of important issues and contributed to the development of new approaches. However, there seems to have been an exaggeration of both the ‘inability’ of RUM to accommodate these behavioural phenomena, as well as the importance of explicitly representing these phenomena (as opposed to approximating their effects). It remains for individual analysts to decide upon the relative merits of different approaches, and specifically to contrast the importance of behavioural realism with the ability to produce outputs that are of practical use, especially for policy analysis. Finally, a key point in our paper is that a model does not necessarily need to ‘represent’ a behavioural process in order for it to demonstrate good performance on data associated with that process.

## Footnotes

1 Founded upon value or subjective judgements about how an economic agent *should* behave.

2 The paradigm of utility maximisation also forms the basis of much of the work on continuous and discrete-continuous choices, but the present paper focusses on discrete choice alone.

3 Founded upon description and explanation of how an economic agent *actually* behaves.

4 Most often, the condition of ‘independence’ of the utility *difference* is achieved by making all the utilities independent. However, the more precise condition on utility differences is important in obtaining appropriate conditions for reference-dependent models.

5
Hargreaves Heap (1992) defined ‘instrumental rationality’ as ‘...the choice of actions which best satisfy a person’s objectives’ (p4).
